# Quality of core collections for effective utilisation of genetic resources review, discussion and interpretation

**DOI:** 10.1007/s00122-012-1971-y

**Published:** 2012-09-15

**Authors:** T. L. Odong, J. Jansen, F. A. van Eeuwijk, T. J. L. van Hintum

**Affiliations:** 1 Biometris, Wageningen University and Research Centre, P.O. Box 100, 6700 AC Wageningen, The Netherlands; 2 Centre for Genetic Resources, The Netherlands (CGN), Wageningen, The Netherlands

## Abstract

Definition of clear criteria for evaluation of the quality of core collections is a prerequisite for selecting high-quality cores. However, a critical examination of the different methods used in literature, for evaluating the quality of core collections, shows that there are no clear guidelines on the choices of quality evaluation criteria and as a result, inappropriate analyses are sometimes made leading to false conclusions being drawn regarding the quality of core collections and the methods to select such core collections. The choice of criteria for evaluating core collections appears to be based mainly on the fact that those criteria have been used in earlier publications rather than on the actual objectives of the core collection. In this study, we provide insight into different criteria used for evaluating core collections. We also discussed different types of core collections and related each type of core collection to their respective evaluation criteria. Two new criteria based on genetic distance are introduced. The consequences of the different evaluation criteria are illustrated using simulated and experimental data. We strongly recommend the use of the distance-based criteria since they not only allow the simultaneous evaluation of all variables describing the accessions, but they also provide intuitive and interpretable criteria, as compared with the univariate criteria generally used for the evaluation of core collections. Our findings will provide genebank curators and researchers with possibilities to make informed choices when creating, comparing and using core collections.

**Electronic supplementary material:**

The online version of this article (doi:10.1007/s00122-012-1971-y) contains supplementary material, which is available to authorized users.

## Introduction

Ex-situ germplasm collections have increased enormously in number and size over the last three to four decades as a result of global efforts to conserve plant genetic resources for food and agriculture. The large sizes of many of these collections, either individually or collectively for a given species complicate the characterisation, evaluation, utilisation and maintenance of the conserved germplasm. The approach of forming core collections was introduced to increase the efficiency of characterisation and utilisation of collections stored in the genebanks, while preserving as much as possible the genetic diversity of the entire collection (Frankel 1984; Brown 1989). Frankel (1984) defined a core collection as a limited set of accessions representing, with minimum repetitiveness, the genetic diversity of a crop species and its wild relatives. From the original definition, several operational definitions have been coined (see Brown 1995 and van Hintum et al. 2000).

Core collections have many roles to play in the management and use of genetic resources. Genebank curators have the responsibility for conservation, regeneration, safety duplication, documentation, evaluation and characterisation as well as facilitating access to the genetic resources in their collections. These activities often require them to make choices or to set priorities among accessions because of limited resources (Brown 1995). Because a core collection is smaller in size compared to the whole collection, it enables some operations of the genebank, such as evaluation (of the selected accessions), to be handled more efficiently and effectively. The reduced size of a core collection is a key to its manageability and, in many cases the representation of the total collection’s diversity enables the core to function as a reference set of accessions for the whole collection (Brown and Spillane 1999).

Since the inception of the idea of core collections over two decades ago, a body of literature on the theory and practice of core collections has accumulated. Very many approaches for selecting core collections have been proposed and used (e.g. M-Strat (Gouesnard et al. 2001), Genetic distance sampling (Jansen and van Hintum 2007), PowerCore (Kim et al. 2007) and CoreHunter (Thachuk et al. 2009)). In comparing the options for assembling a core collection, one of the challenges is to decide on the evaluation criteria for the quality of the result. Various criteria for determining the quality of a core collection have been suggested in the literature, yet very little attention has been given to the analysis of these quality criteria. In fact every researcher appears to have his/her own criteria for the evaluation of core collections.

Thus there is a need to clearly define criteria for the evaluation of the quality of core collections and to relate the different types of core collections to those criteria. For example, a core collection formed for the purpose of capturing accessions with rare or extreme values of the desired trait(s) (e.g. high resistance to pest or high yield) should be evaluated differently from one formed with the intention of representing the (pattern of) genetic diversity in the collection. By the pattern of genetic diversity we refer to the genetic differences among all the accessions which have been accumulated as a result of natural processes, species’ characteristics and historical events.

The debate whether to have a single or several core collections for a given genebank collection is an old but still an interesting one (see Mackay 1995). The initial idea behind a core collection favoured the creation of a fixed core collection, possibly modified in time to accommodate new knowledge and new diversity (Brown 1989). However, there is evidence from the literature to suggest that genebanks are creating different core collections to represent specific sections of their germplasm collections, e.g. Chilean bean core collections (Paredes et al. 2010) and Iberian peninsula common bean core collections (Rodino et al. 2003) and to cater for specific projects. As pointed out by Mackay (1995), to support better the use of available germplasm, sets of diverse accessions need to be established with different selection criteria in mind. This idea is best captured by a computer programme “core selector” developed at Centre for Genetic Resource, The Netherlands (CGN), where a user is allowed to select online a maximum diversity subset of accessions that meets his/her selection criteria (van Hintum 1999). These selections could be considered core collections since they are representative of the genetic variation of a larger group of germplasm accessions. This concept of objective driven core collection deviates from the original idea of the core collection. Brown (1995) recommended that such objective driven subsets of accessions should have name tags that indicate their purposes rather than call them core (e.g. acid tolerant set). Irrespective of the name, it is clear from literature that these objective driven diverse selections are quite popular. Recent developments in computer science, molecular biology and biochemistry suggest that the generation, storage and processing of data from germplasm will cease to be a limiting factor when creating diverse selections.

It should be noted that we are in no way suggesting that the fixed core collection no longer has merits; the compilation of information on representative samples of a given collection still adds value to all accessions. The mini-core collections and reference sets (Odong et al. 2011b; Upadhyaya et al. 2009) as initiated by the Generation Challenge Program of the CGIAR are good examples of core collections serving that purpose. It is important to note that irrespective of the type of core collections, appropriate optimisation and evaluation criteria should be used in creating and evaluating these selections.

In this paper, we will (1) discuss the different types of core collections and proposed criteria appropriate for quality evaluation of each type of core collection; (2) discuss the different criteria used in the literature for evaluating the quality of core collections and relate each criterion to the different types of core collections; (3) use real data sets (molecular marker data) to illustrate the performance of the proposed quality evaluation criteria with respect to the different types (and purposes) of core collections. The outcome of our study will allow researchers and curators to make informed choices from a set of alternative approaches.

### What is a good core collection?

One of the key goals of defining a core collection is the efficient utilisation of available genetic resources and this is best achieved by having clear objectives in mind when selecting entries for the core (Mackay 1995). The answer to the question “what is a good core collection” therefore depends on the objectives for making the core. This can be “conserving as much variation (phenotypic or genotypic) as possible in as few as possible accessions” or “optimising the chance of finding a new allele”. A second question is how to measure quality of the core collection, and this will depend on the type of data available for evaluation.

According to Brown (1989), a good core collection should have no redundant entries (an entry is an accession included in the core), represent the whole collection with regards to species, subspecies and geographical regions and should be small enough to be easily managed. It was suggested by Marita et al. (2000) that core collections can be created with two general purposes in mind (1) maximising the total genetic diversity in a core (as sometimes favoured by taxonomists, and geneticists as well as genebank curators) and (2) maximising the representativeness of the genetic diversity of the whole collection in a core collection (as sometimes favoured by plant breeders). Accordingly, maximising the representativeness of genetic diversity implies also the inclusion of broadly adapted and heterotic materials containing ‘generalist’ alleles in a core collection. Earlier, Galwey (1995) stated the above two purposes of core collections in a slightly different way as: (1) maximising the representativeness of the full range of variation present in the whole collection; (2) maximising the representativeness of the pattern of variation present in the whole collection.

There is also an aspect of balance between representing total diversity and the usefulness of the core to the intended user (Brown 1995). This can be illustrated with some examples. If a breeder searches for accessions with a specific trait of interest (e.g. acid tolerance), it is likely that the best core collection should contain relatively more material from the primary genepool (see Harlan and de Wet 1971 for description of different types of genepools) as compared to the secondary or tertiary genepool, irrespective of the amount of diversity within the primary genepool since there will be a strong preference for material in an adapted genetic background. Although the chances of getting a rare allele might be higher in the secondary or tertiary genepool compared to primary genepool, it is probably cheaper to evaluate more accessions from the primary genepool than to use material from the tertiary or secondary genepool in the breeding program. If a core collection is created in the search for new resistances, the part of the whole collection originating for example from area(s) that in the past had shown to contain resistances should obviously be overrepresented. This implies that the user is often not primarily interested in maximising diversity per se (which could result in core collections with mainly wild and exotic material), but rather in optimising the chance of finding accessions that he/she is looking for as material which is relatively easy to use in for instance a breeding programme. To achieve this, the selection of a core collection often starts with stratifying accessions into homogeneous groups (a group can be collection of accessions with similar characteristics, e.g. phenotype, genotypes or region of origin), followed by an arbitrary determination of the number of accessions to be selected from each group, the so-called allocation. When a core collection is being formed for a specific user, the stratification and allocation processes can be used to ensure that accessions from (a) particular group(s) (e.g. primary gene pool, modern varieties or Ethiopian landraces) are given more priority than justified by the genetic variation contained in that group. Since each user or curator most often define their own methods for stratification (dividing accessions into groups) and for determining the number of accessions to select from each group, it is difficult to setup uniform criteria for evaluating those objective driven core collections.

From the literature, it is not clear how to relate the purpose of the core collections with the various quality evaluation criteria, and only very few authors have attempted this (e.g. Thachuk et al. 2009). Based on the purposes of core collections as suggested by Galwey (1995) and Marita et al. (2000), we have identified three broad types of core collection which will be discussed in the next section. We relate each of the three types of core collections with their respective evaluation criteria.

## Types of core collections

Based on the purposes for which they are formed core collections can generally be classified into three types or categories (i.e. core collections representing (1) individual accessions; (2) extremes; and (3) distribution of accessions in the whole collection). In defining the types of core collections, the term “accessions” refers to elements that constitute the whole collection and “entries” are elements of the core collection. Since the core collection is a selection from the whole collection, all entries are accessions, but only few accessions are entries.

### Type 1

A core collection representing the individual accessions of the whole collection (CC–I). In this case each entry in the core collection represents one (itself) or more accessions that jointly make up the whole collection. Each accession in the whole collection is represented by an entry in the core which is most similar to it.

**Fig. 1:**
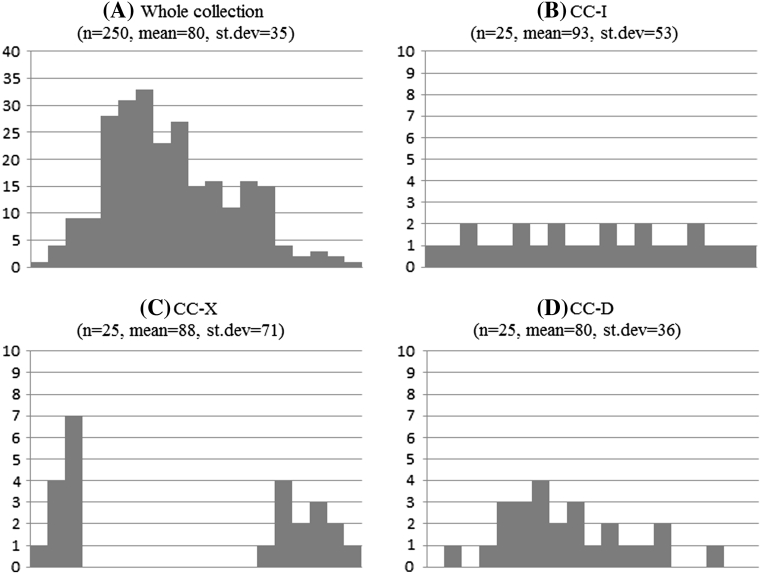
**a** Multimodal trait distribution of for whole collection; **b** distribution of the same trait for a collection of type CC–I; **c** distribution of the same trait for a core collection of type CC–X; **d** distribution of the same distribution for a collection of type CC–D

This type of core collection (CC–I) aims at a uniform representation of the original genetic space, with equal weights across this space and is the most intuitive way of looking at core collection (see Fig. 1). A core collection of type CC–I is especially suitable, for situations requiring an overview of the genetic diversity of the accessions of the whole collection. Core collections formed for the purposes of maximising the representativeness of genetic diversity as suggested by Marita et al. (2000) can be placed in type CC–I. By ensuring that all accessions in the entire collection are maximally represented, core collections of type CC–I provide the best option for obtaining a single “multi-purpose” or generalist core collections compared to any type of core collection.

### Type 2

A core collection representing the extremes of the whole collection (CC–X). *Implication* the diversity of the traits of the entries of the core collection is maximised.

A core collection of type CC–X is geared towards representing the ranges of phenotypes, genotypes or alleles of the whole collection. A good core collection of type CC–X has entries that are as different as possible from each other. A core collection representing the total genetic diversity, as suggested by Marita et al. (2000), can be considered as a core collection of type CC–X.

### Type 3

A core collection representing the distribution of accessions of the whole collection (CC–D). In this case, when creating a core collection one ensures that the proportion of accessions in that core collection reflects the numerical contributions of the different regions or categories to the whole collection. For example, if the majority of the accessions come from a given geographical region, then the core collection should adequately reflect the importance of that region.

*Implication* the distributions of all relevant traits over the entries of the core are similar (in terms of mean, variance, quartiles, frequencies) to those of the whole collection.

In our opinion, this core collection of type CC–D is only of interest if the aim is to give an overview of the composition of the whole collection using only a part of the collection. This type of core collection will be obtained by maximising the representativeness of the pattern of variation of traits in the whole collection, as suggested by Galwey (1995).

Based on the criteria used for the evaluation of core collections in literature, it appears that either most of the core collections are intended to be of type CC–D or they were evaluated with inappropriate criteria (Diwan et al. 1994), sesame core collection: China (Xiurong et. al 2000), Iberia Peninsula common bean (Rodino et al. 2003), groundnut (Upadhyaya 2003), peanut (Valencia) (Dwivedi et al. 2008), USDA soybean core (Oliveira et al. 2010). This could be an indication of the desire of researchers to have a single “multi-purpose” core collection from which one could extract materials for different purposes. It should be noted that by insisting on selecting a core collection that reproduces the distribution of traits in the whole collection one ignores the issue of redundancies and over representation. As we stated earlier, for researchers aiming at a “multi-purpose” core collection the CC–I type of core collection would be the much better option compared to the CC–D type.

The different types of core collections have been illustrated graphically using a multimodal univariate distribution for the whole collection (Fig. 1).

### Quality criteria for evaluating core collections

The process of evaluating a core collection usually involves a comparison with the whole collection from which it has been obtained, or a comparison with alternative core collections (core collected created using different methods). This requires clear and objective criteria for assessing the quality of the different types of core collections.

Irrespective of the type of core collection and the quality criterion used, the evaluation of quality should be based if possible on data (traits or characteristics) that were not used in the selection of the core (van Hintum et al. 2000). This might sound like an obvious statement, but it is very often neglected (e.g. Tai and Miller 2000; Wang et al. 2007). For example, one has a dataset of 1,000 accessions each genotyped with 50 markers, and the objective is to create a core collection of 20 entries with maximal allelic richness. If it would concern only the current 50 markers, this would be a simple optimisation problem. However, the question is, “what if the core collection should also be ‘allelic rich’ for all loci that were not genotyped?” One option would be to use half the markers for creating core collections, and the other half for evaluating the quality of the resulting core collection(s) (for a good examples see Mckhann et al. (2004); Ronfort et al. (2006); Balfourier et al. (2007)). Once the best strategy has been determined this strategy could then be used on the entire set of markers to create the final core collection. Since often molecular data will be used to select a core that is also supposed to optimise the representation of phenotypic diversity, relevant phenotypic traits should be used for the validation as well.

In this article, we place emphasis on evaluation criteria that are based on genetic distances between accessions. The main advantage of using genetic distance for evaluation of core collections is that unlike the other criteria used in literature which handle one variable at a time, all the variables are used simultaneously. It is also easier and more intuitive to link distances to the concept of genetic diversity.

### Evaluation of type CC–I

**Fig. 2:**
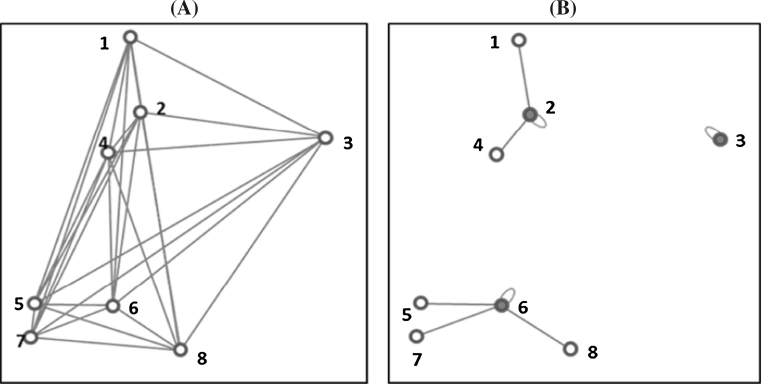
**a** Eight accessions (1, 2, …, 8) in a 2D space with all pairwise distances (the distance between accession *i* and *j* is indicated as *D*_*i*−*j*_). **b** The three selected entries (highlighted accessions) based on the A–NE criterion, minimising the average distance between each accession and it nearest neighbouring entry (*D*_1−2_ + *D*_2−2_ + *D*_3−3_ + *D*_4−2_ + *D*_5−6_ + *D*_6−6_ + *D*_7−6_ + *D*_8−6_)/8

A good criterion for the evaluation of a CC–I core should be able indicate how well each accession in the whole collection is represented in the core collection. This involves establishing the relationships between each accession in the whole collection with the entries of the core collection. The relationship between accessions and entries is best represented by genetic distances between. For CC–I, we proposed a criteria based on distances between each accession in the whole collection and the nearest entry in the core collection (A–NE) (see Fig. 2a, b).

#### Average distance between each accession and the nearest entry (A–NE) (Odong et al. 2011b)

In this case, the distance between each accession and the nearest entry in the core is calculated and averaged over all the accessions. For the selected accessions (entries) these distances are taken as zero (they are closest to themselves). For example, the value A–NE for Fig. 2 is given as:

$$ {\text{A}{-}\text{NE}} = \frac{{(D_{1 - 2} + D_{2 - 2} + D_{3 - 3} + D_{4 - 2} + D_{5 - 6} + D_{6 - 6} + D_{7 - 6} + D_{8 - 6} )}}{8} $$

where $D_{i - j}$ (*i, j* = 1, 2, …, *n*; *n* is the number of accessions in the whole collection).

For core collections of type CC–I, the value of A–NE should be as small as possible; the maximum representation (A–NE = 0) is obtained when each accession is represented by itself or by an identical duplicate accession in the core. In core collections that optimise the values of A–NE (CC–I type of core), the accessions selected as entries tend to be those at the centres of clusters (i.e. groups) rather accessions on the outer layer of the clusters.

### Evaluation of type CC–X

A good criterion for a core collection of type CC–X (representing the extreme values) should be able to quantify differences between entries of the core collection as well as being able to measure the inclusion or exclusion of accessions with extreme values of the relevant traits in the core. The most intuitive criteria for determining differences between entries in the core collection are those criteria based on pairwise distances. The exclusion or inclusion of accessions with extremes values in the core can be assessed using frequencies of traits or alleles captured (see Thachuk et al. 2009). Below we propose a new criterion based on distances between an entry and the nearest neighbouring entry (E–NE) and compare it with criteria based on average pairwise distances between all entries.

#### Average distance between each entry and the nearest neighbouring entry (E–NE)

**Fig. 3:**
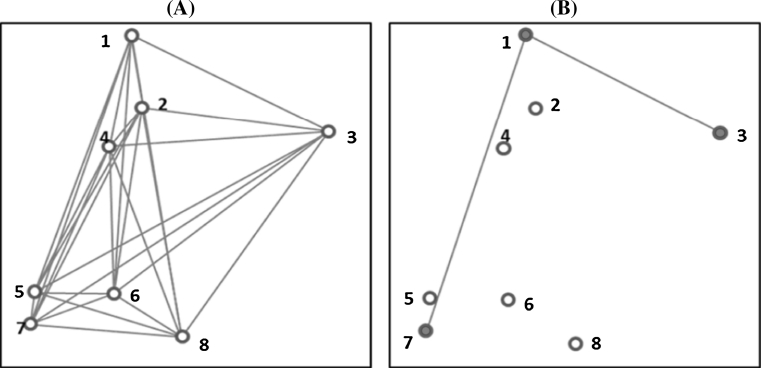
**a** Eight accessions (1, 2, …, 8) in a 2D space with all pairwise distances (the distance between accession *i* and *j* is indicated as *D*_*i*−*j*_). **b** The three selected entries (highlighted accessions) based on the E–NE criterion maximising distances between each entry and the nearest neighbouring (*D*_1−3_ + *D*_3−1_ + *D*_7−1_)/3

**Fig. 4:**
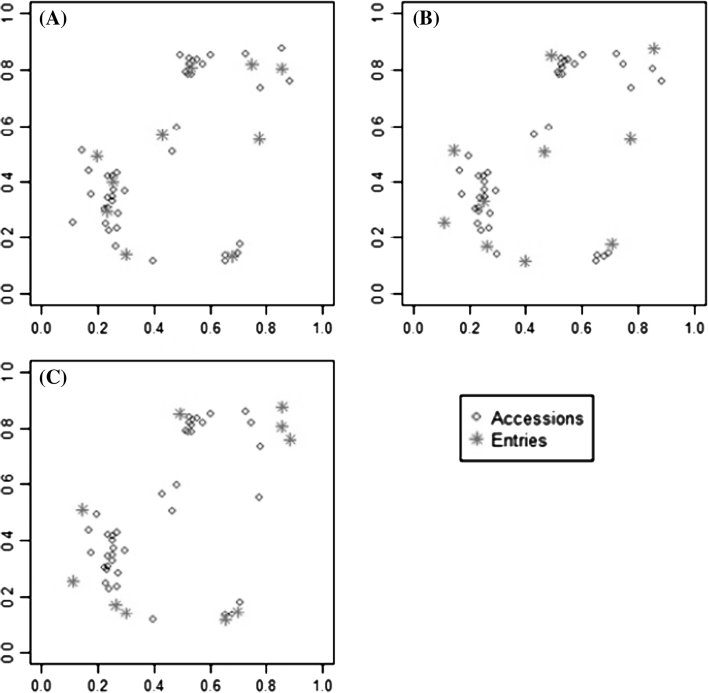
Examples of core collections, showing the effect of optimisation of different criteria on the positioning of entries (*red stars*) within the distribution of accessions (*circle*) for each core collection, the value of all three evaluation criteria are given: **a** the average distance between each accession and the nearest entry (A–NE) is minimised (E–E = 0.467; E–NE = 0.180; A–NE = 0.038) **b** the average distance between an entry and the nearest other entry (E–NE) is maximised (E–E = 0.491; E–NE = 0.241; A–NE = 0.056) **c** the average distance between entries (E–E) is maximised (E–E = 0.573; E–NE = 0.118; A–NE = 0.094). Thus, for E–E and E–NE, the larger the value the higher the quality of the core collection, the opposite is true for A–NE

According to this criterion (E–NE), a good core collection is one that maximises the average distance between each entry and the nearest neighbouring entry in the core collection. For this criterion, each entry should be as different as possible from each other. This avoids selecting a few clusters of similar accessions at the extreme ends of the distribution, that might occur if one chooses a set of entries that maximises the average of all pairwise distances between the entries in the core (E–E) (see Fig. 4). When calculating E–NE only a subset of pairwise distances between the entries are used. Using example in Fig. 3, if accessions 1, 3 and 7 are selected as entries in the core collection, and if we assume that; (i) entry 1 is the nearest neighbouring entry to both 3 and 7 ($D_{3 - 1} < D_{3 - 7}$ and $D_{7 - 1} < D_{7 - 3}$); and (ii) entry 3 is the nearest neighbour to entry 1 ($D_{1 - 3} < D_{1 - 7}$) then E–NE is given as:

$$ {\text{E}{-}\text{NE}} = \frac{{(D_{1 - 3} + D_{3 - 1} + D_{7 - 1} )}}{3} $$

where $D_{i - j}$ (*i, j* = 1, 2, …, *n*; *n* is the number of accessions in the whole collection)

#### Average genetic distances between entries (E–E)

Maximising the average genetic distance between entries of a core collection has been suggested as a desired quality criterion for evaluating core collections intended for plant breeders (Franco 2006, Thachuk et al. 2009). Using example in Fig. 3, E–E are given as:

$$ {\text{E}{-}\text{E}} = \frac{{(D_{1 - 3} + D_{1 - 7} + D_{3 - 7} )}}{3} $$

where $D_{i - j}$ (*i, j* = 1, 2, …, *n*; *n* is the number of accessions in the whole collection).

Figure 4 provides a simple numeric and graphical comparisons of the three distance-based criteria discussed above. Although both E–E and E–NE are suitable for the CC–X type of core, as illustrated in Fig. 4c core collections with a high average distance between the entries (E–E) can still have a high level of redundancies. It is clear from Fig. 4 that despite having the highest E–E (0.573 vs. 0.491 and 0.467) the core collection in Fig. 4c, some entries in Fig. 4c are too close to each other to be included in a core collection as reflected by a low value of E–NE. Figure 4a indicates that minimisation of A–NE leads to the selection of accessions from the centres of clusters compared to E–E and E–NE which select accession at the periphery of clusters.

### Evaluation of type CC–D

Ideal criteria for evaluating a core collection of type CC–D should be able to compare many distributional aspects simultaneously: centre (mean, mode), spread (variance, range), shape (symmetry, skewness, number of modes) and unusual features (gaps, presence of outliers) of all data simultaneously. For continuous data, we propose the use of quantile–quantile plots (Gnanadesikan and Wilks 1968) which provide a visual comparison for two data sets using several distributional aspects of the data simultaneously. We also recommend the use of Kullback–Leibler distance (Kullback and Leibler 1951) which measures the distance between probability distributions and can be used to compare the difference in probability distribution between the core collection and the whole collection. A brief description of Kullback–Leibler distance is presented in Electronic Supplementary Material (Appendix 1).

#### QQ plot

**Fig. 5:**
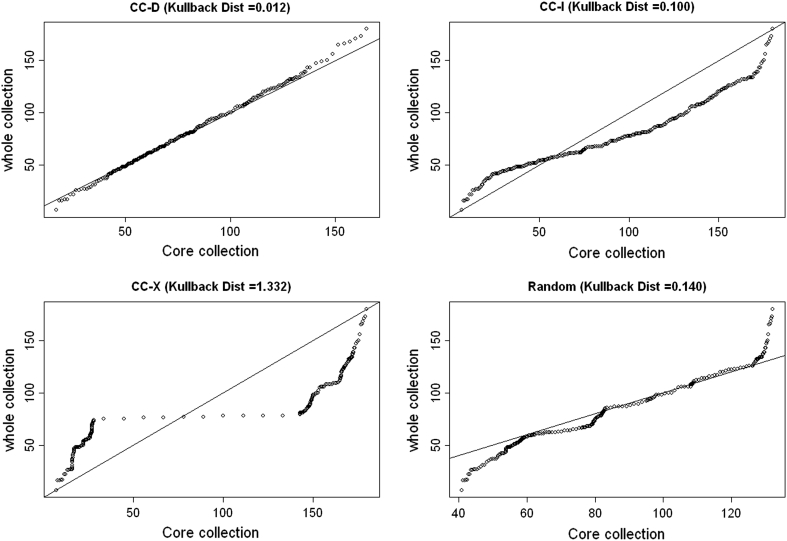
QQ plots for different types of core collections shown in Fig. 1. From both the QQ plots and Kullback distance, it is clear that the distribution of whole collection is best represented by type 1 (CC–D) core. The Kullback–Leibler distance (Kullback Dist) was calculated based on values generated by the QQ plot. Random sampling core collection is only based on 1 data set. The minimum value of Kullback–Leibler distance is zero (for a core collection with identical distribution to that of the whole collection)

Compared to simple comparison of means or variances the QQ plot gives a much better overall visual view of how the distribution of a given trait differs between the core collection and the whole collection. A QQ plot is a graphical method for comparing two probability distributions by plotting corresponding quantiles against each other. If the two distributions are similar, the points in the QQ plot will lie approximately on a straight line. A QQ plot is generally a more powerful approach for comparing distributions than the common technique of comparing histograms of the two samples, but requires more skills for correct interpretation. A more quantitative approach for comparing the distribution of the traits in the whole collection and the core would be to calculate the Kullback–Leibler distance between the core collection and the whole collection. Figure 5 shows QQ plots for the three core collections types shown in Fig. 1. We have also used the information from QQ plot to calculate the Kullback–Leibler distance between the different core collections in Fig. 1 and the whole collection.

### Common methods used for evaluating core collections in the literature

Below we give an overview of the various criteria for evaluating core collections used in the literature and relate them to the three types of core collection. Given that the type of data determines how diversity in the whole collection or the core collection should be quantified, we will also try to relate the evaluation criteria to the different types of data (see Table 1 for brief descriptions of different types of data that are being used for selecting and evaluating the quality of core collections). It should be noted that when evaluating the quality of core collections, most authors apply several evaluation criteria despite the fact that those criteria are only suitable for specific aspects of core collections. The most common criteria used for evaluating core collections include criteria based on summary statistics, the Shannon diversity index, class/category coverage and Chi-square tests of association (see Table 2 below for summary).

**Table 1:** Brief description of of data types used for creating and evaluating core collections

Several types of information can be used for selecting core collections. The most common type of data are (i) passport data (ii) agronomic data and (iii) molecular marker data Passport data Passport data are data about the identity and origin of an accession, including its taxonomic classification, with connected knowledge about domestication, distribution, breeding history, cropping pattern and utilisation. Example of passport data include the country of origin, the crop type (e.g. winter or summer wheat), and pedigree Agronomic data Agronomic data can be continuous, discrete or categorical. Examples of continuous variables include grain yield, plant height, leaf area, etc. Discrete variables deal with counts such as the number of fruits or the number of seeds in a pod. Categorical variables may be defined as binary (presence or absence of a given characteristic), nominal (colour or shape of an organ) or ordinal (a visual scale arranged to represent intensity, colour or size) (Crossa and Franco 2004). Agronomic traits are usually controlled by multiple genes and typically by environmental factors Molecular data Data from molecular or biochemical marker systems can be treated as either continuous (allele frequency) or categorical (presence or absence of band or allele). Examples of popular molecular data types include those generated by single nucleotide polymorphism (SNP), amplified fragment polymorphism (AFLP), random amplified polymorphic DNA (RAPD), and simple sequence repeats (SSR)

**Table 2:** Summary of common methods (criteria) used for evaluation of the quality of core collections in literature

Criteria	Type of variables	General comments
Summary statistics	Continuous	Compare the mean, variance, etc. of the core with that of whole collection Comparison is done for one variable at a time and later combined Mainly suitable for CC–D type of core collections
Principal component analysis	Continuous	Plot of the coordinates of the entries on the main principal components (exploratory) to show spatial distribution of entries and accessions Compare two core collections using sum of squares of the their scores along the major PCs (Suitable for CC–X core type)
Shannon diversity Index (SH)^b^	Categorical	The highest value is obtained when all the categories in the whole collection are represented in equal proportion (penalizes redundancy at the category level) The value of SH of a given core collection should be compared with the maximum possible value (log (*n*), where *n* is the number of classes in the whole collection) Most authors apply this criterion inappropriately by comparing SH value of the core collection with that of the whole collection Suitable for CC–I core type
Class coverage^b^	Categorical	The highest value (1 or 100 %) is obtained when all the categories in the whole collection represented in the core Unlike SH it does not correct for redundancy in the core collection Suitable for CC–I core type
Chi-square goodness-of-fit^b^	Categorical	This criterion has been used to test for the deviation of the frequency distributions of important categorical traits between core collection and the whole collection A good core collection is one in which the frequency distribution of the categories of the core is not statistically different from that of the whole collection Suitable for CC–D core type

^a^For all criteria except Principal component, the criterion is calculated for each variable at a time and later combined

^b^Can be applied to ccontinuous variables by first putting values into specific number of classes (determining the number classes is challenging)

#### Summary statistics

Criteria based on mean, variance and other summary statistics such as coefficient of variation, range, inter-quartile range have been used mainly to evaluate the quality of core collections based on continuous traits (Hu et al. 2000; Tai and Miller 2000). It involves statistical tests of differences between means, variances and other summary statistics of the core and the whole collection. Based on the results of statistical tests (mainly *t* tests and F tests) performed on each trait separately, several evaluation criteria (mean difference percentage, variance difference percentage, coincidence rate of change and variable rate of coefficient of variation, sign test) have been suggested (see Table 3). Criteria based on means and variances are probably suitable for the evaluation of a core collection of type CC–D and will perform very poorly with core collections of types CC–I and CC–X.

**Table 3:** Common criteria for evaluating the quality of core collections based on summary statistics

Criteria	Description
Mean difference percentage (MD) (Hu et al. 2000 ^a^)	${\text{MD}} = \left( {\frac{{{\text{S}}_{t} }}{n}} \right) \times 100$ where *S*_*t*_ is the number of traits with a significant difference between the means of the whole collection and the core collection; $n$ is the total number of traits. The lower (<20 %) the value of MD the more representative the core collection
Variance difference percentage (VD) (Hu et al. 2000)	$VD = \left( {\frac{{S_{t} }}{n}} \right) \times 100$ where *S*_*t*_ is the number of traits with a significant difference between the variances of the whole collection and the core collection; $n$ is the total number of traits. The larger (>80 %) the value of VD, the more diverse the core collection
Coincidence rate of range (CR) (Diwan et al. 1995)	$CR = \frac{1}{n}\sum\limits_{i = 1}^{n} {\frac{{R_{C(i)} }}{{R_{W(i)} }}} \times 100$ where $R_{C(i)}$ and $R_{W(i)}$ represent the ranges of the* i*th trait in the core collection and the whole collection, respectively; $n$ is the total number of traits
Variable rate of coefficient of variation (VR) (Hu et al. 2000)	$VR = \frac{1}{n}\sum\limits_{i = 1}^{n} {\frac{{CV_{C(i)} }}{{CV_{W(i)} }}} \times 100$, where $CV_{C(i)}$ and $CV_{W(i)}$ represent the coefficients of variation of the $i \rm{th}$ trait in the core collection and the whole collection, respectively; *n* is the total number of traits
The Sign (+ versus −) test (Basigalup et al. 1995, Tai and Miller 2000)	$X^{2} = \left( {N_{1} - N_{2} } \right)^{2} /\left( {N_{1} + N_{2} } \right)$. where *N*_1_ is the number of variables for which the mean or variance of the core collection is greater than the mean or variance of the whole collection (number of + signs); *N*_*2*_ is the number of variables for which the mean or variance of the core collection is less than the mean or variance of the whole collection (number of − signs). The values of *X* ^2^ should be compared with a Chi-square distribution with 1 degree of freedom

^a^For a core collection to be representative of the whole collection, the value of MD should not be more than 20 % and the value of CR should be greater than 80 % (Hu et al. 2000)

Some authors have questioned the use of differences between means and variances of core and whole collection as criteria for evaluating the quality of core collections (e.g. Kim et al. 2007). There is also a conceptual problem when comparing a core collection and a whole collection. Statistically a core collection is a sample from the whole collection (i.e. a population). Thus the question is not whether these two samples are different, but could this sample (core collection) have come from this population distribution (i.e. the whole collection)? So we should be dealing with a one-sample test and not a two-sample test. It is thus clear that the use of QQ plot (Gnanadesikan and Wilks 1968) and probability distribution based methods such as the Kullback–Leibler distance (Kullback and Leibler 1951) would be the best option for evaluation of CC–D types of core collections.

Apart from the criteria described in Table 3, the phenotypic correlation coefficient of different traits has also been used as a criterion for evaluating the quality of core collections (Reddy et al. 2005; Mahajan et al. 2007). The pairwise phenotypic correlation coefficients between different traits are calculated separately for the core collection and whole collection and the values are then compared in order to determine whether the associations between traits have between conserved well enough in the core collection.

#### Principal component analysis

Another exploratory criterion for evaluating core collections involves the inspection of the spatial distribution of the entries in plots of principal components (Bisht et al. 1998; Kang et al. 2006, Mahajan et al. 2007). Based on the method suggested by Noirot et al. (1996), it is possible to compare two core collections or relate the core collection with the whole collection based on the sum of squares of the scores of the entries on the major principal components: the greater the value, the more diverse the core collection. This criterion would be suitable for evaluation of core collections of type CC–X. However, it should be noted that a core with a higher value for this criterion can still have a high level of redundancy resulting from the inclusion of two or more similar accessions from the extreme ends of the distribution.

#### Shannon diversity Index (SH)

This criterion is suitable for evaluating core collections using categorical data; it has been used extensively in the literature. For a given trait, the Shannon diversity index (Shannon 1948) is calculated as follows:

$$ {\text{SH}} = - \sum\limits_{i = 1}^{n} {p_{i} } \log (p_{i} ) $$

where $p_{i}$ is the frequency of the category $i$ and *n* is the total number of categories. The SH penalizes redundancy at the category level and its maximum value (log(*n*)) is obtained when all classes are represented in equal proportions (i.e. $p_{1} = p_{2} = \cdots = p_{n} = 1/n$). Therefore, in terms of SH, the best core collection should be the one with the maximum attainable value which makes SH a suitable criterion for core collections of type CC–I. Note that the whole collection will never attain the maximum possible value of SH because of redundancy associated with it. A core collection should be expected to have higher SH values as compared to the whole collection. Similarity of these SH values is not an indication of a good core collection, contrary to what is often concluded in the literature (e.g. Bisht et al. 1998; Upadhyaya 2003; Mahalakshmi et al. 2007; Dwivedi et al. 2008; Upadhyaya et al. 2009).

To apply SH or other measures of diversity to continuous agronomic data, the data should first be converted into categorical data by putting them into a specific number of classes.

#### Class coverage (Coverage)

This criterion reports the percentage or proportion of the categories in the whole collection that have been retained in a core collection (Kim et al. 2007). It is defined by:

$$ {\text{Coverage}} = \left( {\frac{1}{K}\sum\limits_{k = 1}^{K} {\frac{{A_{\text{Core}} }}{{A_{\text{Wcol}} }}} } \right) \times 100 $$

where $A_{\text{Core}}$ is the sets of categories in the core collection and $A_{\text{Wcol}}$ is the sets of classes found in the whole collection and *K* is the number of traits. According to this criterion, a good core collection should retain all categories of a given variable in the whole collection. For the case of molecular marker data, the categories represent the number of distinct alleles (akin to allelic richness) in the whole collection. Class coverage is also a suitable quality criterion for core collections formed with the purpose of adequately representing the accessions in the whole collection (type CC–I). When this criterion is applied to molecular markers it will be suitable for core collections aimed at capturing accessions with rare alleles (type CC–X).

It should be noted that unlike SH, coverage does not take into consideration the differences in frequency of the categories represented in the core collection so a core collection with high coverage can still have a high level of redundancy. Just like with SH, deciding on the number of categories (intervals for continuous data) is a major challenge when calculating coverage.

#### Chi-square goodness-of-fit

This criterion has been used to test for the deviation of the frequency distributions of important categorical traits between core collection and the whole collection (Tai and Miller 2000; Grenier et al. 2000; Zeuli and Qualset 1993). Chi-square goodness-of-fit can also be used for continuous agronomic data converted into categorical data. The Chi-square values can be computed as:

$$ \chi^{2} = \sum\limits_{i = 1}^{k} {\frac{{({\text{CFreq}}_{i} - {\text{WCFreq}}_{i} )^{2} }}{{({\text{WCFreq}}_{i} )}}} $$

where ${\text{CFreq}}_{i}$ is the relative frequency of accession from category $i$ ($i = 1,2, \ldots ,k$) in the core collection and ${\text{WCFreq}}_{i}$ is the relative frequency of accessions from category $i$ in the whole collection. The number of degrees of freedom being the number categories (classes) minus one. This test (Chi-square) is only suitable when the interest is in representing the distribution of traits of accessions in the whole collection (type CC–D).

From the literature, it clear that criteria based on summary statistics and SH are the most frequently used (see Table 4). Since most core collections in literature are evaluated using similar evaluation criteria, one would be tempted to believe that all those cores were obtained with the same objective(s) in mind. We highly doubt whether all those core collections were indeed made with the same objective(s) in mind.

**Table 4:** Examples of Core collections from literature showing data and criteria used for their evaluating them

Paper (Core)	Data use for selection	Data use for evaluation	Criteria use for evaluation
Soybean core collection (Oliveira et al. 2010)	P, A, M	A, M	Summary statistics, Chi-square, correlations
Sorghum mini-core (Upadhyaya et al. 2009)	P, A, M	P, A, M	Summary statistics, Chi-square, SH, correlation
Mini-core Japanese rice landraces (Ebana et al. 2008)	Markers	Markers, A	Percentage of alleles retained, summary statistics
Peanut (Valencia) (Dwivedi et al. 2008)	P, A, M	P, A, M	Summary statistics, Chi-square, SH, correlation
A worldwide bread wheat (Balfourier et al. 2007)	P, Markers	P, Markers^a^	Alleles captured, countries of origins represented
Pearl millet (Bhattacharjee 2007)	P, A, M	P, A, M	Summary statistics, Chi-square, SH, correlation
World sesame (Mahajan et al. 2007)	P, A, M	A, M	Summary statistics, correlations, SH, PCA
West African yam Dioscorea spp. (Mahalakshmi et al. 2007)	P, A, M	A	Summary statistics, correlation, Chi-square, SH
USDA rice (Yan et al. 2007)	P	A, M^a^	Summary statistics, correlation
Korean Sesame core (Kang et al. 2006)	P, A, M	A, M	Summary statistics, Chi-square PCA
Pigeon pea (Reddy et al. 2005)	P, A, M	P, A, M	Summary statistics, Chi-square, SH, correlation
Iberia Peninsula common beans (Rodino et al. 2003)	P	A, M	Summary statistics, Chi-square
Groundnuts (Upadhyaya 2003)	P, M	M	Summary statistics, Chi-square, SH, correlation
Sesame -China (Xiurong et al. 2000)	P, A, M	A, M	Summary statistics
Indian Mung Beans (Bisht et al. 1998)	A, M	M^a^	Summary statistics, PC, SH
Perennial *Medicago* **(**Basigalup et al. 1995)	P, A, M	A, M	Summary statistics
Annual Medicago (Diwan et al. 1994)	P, A, M	P, A, M^a^	Summary statistics

*A* Agronomic data, *M* Morphological data, *P* Passport data, *PCA* Principle component analysis, *SH* Shannon Diversity Index

^a^Part or all the data used for the evaluation was different from the one used for forming the core collection

## Illustration using real data sets

### Description of the datasets

We used two published data sets, i.e. coconut and common bean (Odong et al. 2011a, b) to demonstrate the importance of choosing the right criteria for each type of core collection (see below description of the data for details). In this section we also demonstrate that a core collection which optimises a given criterion in one dataset may not do well when evaluated using another dataset.

#### Coconut (*Cocos nucifera*)

The coconut data consist of 1,014 accessions of coconut accessions genotyped with 30 SSR markers. The accessions were collected from different regions of the world: West Africa (32), North America (52), South Asia (62), Latin America (72), Central America and the Caribbean (109), East Africa (124), South East Asia (183) and the Pacific Islands (380). Coconut is a diploid, mainly out-crossing species. Most of the accessions in this set were indicated as tall; 43 dwarf accessions were present mainly from South East Asia. Dwarf coconuts have a high degree of self-fertilization. Because of its usefulness, coconut has been extensively distributed around the world. For this study, the coconut data were selected because it contained larger numbers of accessions of each of the diverse origins (a typical albeit virtual genebank germplasm collection).

#### Common bean (*Phaseolus vulgaris*)

The common bean data set consisted of 603 accessions with 296 being described as Andean and 307 as Mesoamerican types, genotyped with 36 SSR markers. These accessions originated from 24 different countries with most of them coming from Peru (184), Mexico (178), Guatemala (61), Ecuador (35), Colombia (29) and Brazil (22) and the remaining 18 countries contributed 94 accessions. The common bean is a self-pollinating diploid species. Twenty-nine of the 36 SSR markers used in study belong to known linkage groups.

### Comparing the performance of the evaluation of criteria (A–NE, E–NE and E–E) when applied to different types of core collections

The aim of this subsection is to demonstrate the importance of using appropriate quality evaluation criteria when evaluating the different types of core collection. We created core collections of different sizes (5, 10, 15, …, 100) by optimising (minimising or maximising) each of the three evaluation criteria (A–NE, E–NE and E–E). That is, for each quality evaluation criterion we created 20 core collections of different sizes and each core collection was evaluated using the other two evaluation criteria which were not used for creating it. For example core collections created by maximising the value of E–NE are evaluated using A–NE and E–E criteria. Core collections which minimises A–NE were created using Genetic Distance Optimisation method (Odong et al. 2011b) and those which maximise E–E were created using Corehunter (Thachuk et al. 2009). We wrote an R programme (available on request from the authors) for creating core collections which maximises E–NE. For comparison purposes random sampling was also used to create core collection of the same sample sizes (5, 10, 15, …, 100).

**Fig. 6:**
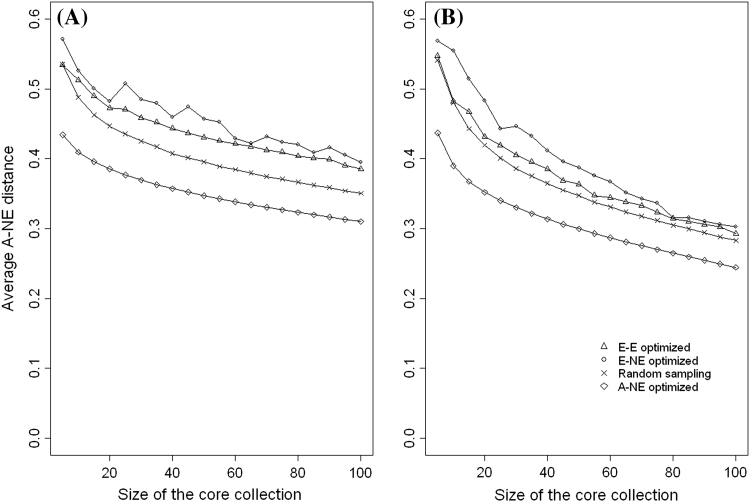
Plot of Average distance between each accessions and its nearest entry in the core (A–NE) against different sizes of collections formed by optimising (minimising or maximising) different criteria (E–E, E–NE, A–NE and Random sampling) using Coconut (**a**) and Common beans (**b**)

**Fig. 7:**
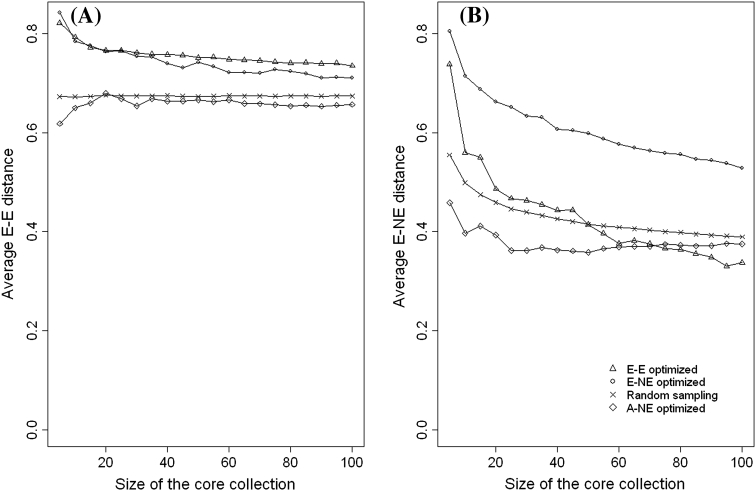
Plot of average distances between the entries in the core collection (E–E) (**a**) and average distance between an entry and the nearest neighbouring entry (E–NE) (**b**) against the size of core collection for cores formed by optimising different criteria (E–E, E–NE, A–NE and random sampling) for Coconut data (1,014 accessions)

**Fig. 8:**
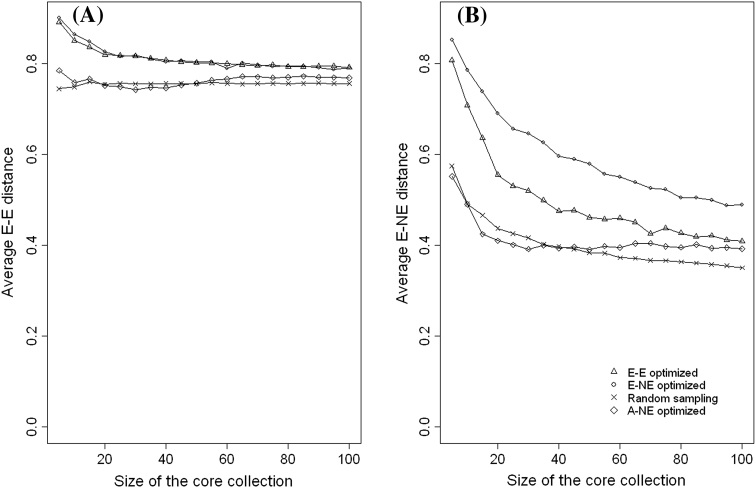
Plot of average distances between the entries in the core collection (E–E) (**a**) and average distance between an entry and the nearest neighbouring entry (E–NE) (**b**) against the size of core collection for cores formed by optimising different criteria (E–E, E–NE, A–NE and random sampling) for Common bean (515 accessions) data

For both coconut and common bean data, Fig. 6 shows that in terms of A–NE (representing accessions in the whole collection), core collections formed by maximisation of E–NE or E–E perform even poorer than random sampling. On the other hand, the performance of core collections formed by minimising A–NE performed poorly when evaluated using E–NE or E–E criteria (see Figs. 7, 8). This shows that when selecting a core collection, it is essential to define the objectives clearly and the objectives should be the basis for choosing the evaluation criteria. It is clear from these two examples that if one evaluates a core collection of type CC–X with inappropriate criteria (e.g. A–NE instead of E–NE or E–E) there is a high likelihood of drawing a wrong conclusion. The poor performance of core collections formed by maximising A–NE (CC–I type of core collection) when evaluated using E–NE and E–E indicates the challenges of constructing a single “multi-purpose” core collection from which one could extract material of interest. However, the poor performance of the core obtained by minimising A–NE when accessed using E–NE or E–E does not mean that such core collections do not have accessions with extreme characteristics. In CC–I type of core collections accessions with extreme characters are those one that represent themselves (i.e. CC–I core put emphasis on both accessions with common and rare traits).

We have shown in Figs. 7 and 8 that for both crops a core collection that maximises E–NE also performs (maximises) very well with respect to E–E but the reverse is not always true (i.e. maximising E–E can result in a much lower value of E–NE since similar accessions at the extreme ends of the distributions can be included in the core). In general, for both coconut and common beans, data sets comparison based on E–E is less responsive to changes within the core collection introduced by either changing the number of entries (5–100) or changes in the optimisation methods used for forming the core collection. For example, for both crops (Figs. 7, 8), the changes in E–E between a core with a size of 5 and a size of 100 ranges between 1.5 and 12 % compared to the changes in E–NE, which lies between 18 and 54 %. The little response of E–E to changes within the core collection is due to the fact that as the core collection size increases, the average distance between entries (E–E) tends towards the overall mean of distances between accessions in the whole collection (the E–E line of random sampling; Figs. 7a, 8a). This is a clear indication that although both E–NE and E–E can be used for evaluating the quality CC–X type of core collection, E–NE appears to be more reliable.

### Use of different data sets for evaluating core collections

In this subsection we show that a core collection obtained by optimising a given criterion using one set of variables (data set) may not be optimal for another set of variables. The evaluation of a core collection with the same data set that was used to create it ignores this simple but very important point. This is quite important, especially in the case of molecular markers data where the key assumption is that by maximising diversity in a given set of marker loci, the diversity of genes of interest will also be maximised. In this study we randomly divided the two datasets into two equal datasets in terms of the number of molecular markers (18 and 15 markers each for bean and coconut datasets, respectively). For each crop, one half of the data was used to form the core collection (*training dataset*) and the other half used for evaluation of the resulting core collection (*evaluation set*). For the *evaluation set* we first determined the maximum (for E–NE) and minimum (A–NE) possible value of the evaluation criteria. We referred to this maximum or minimum possible value attainable from evaluation set as *Target* (E–NE or A–NE), while *Actual* (E–ENE or A–NE) values are obtained when core collections that were created using the core/training set and evaluated using the evaluation set. The core collections formed were of the same samples sizes as those formed in the previous subsection (5, 10, 15, …, 100). Randomly generated core collections of the same sizes were also evaluated.

**Fig. 9:**
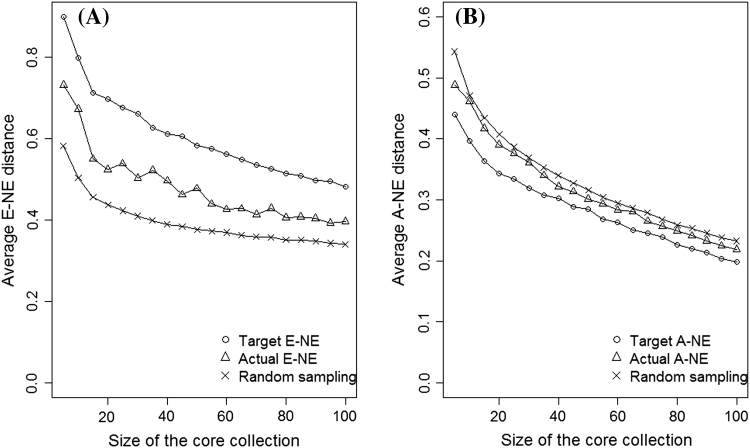
Plot of average distance between an entry and the nearest neighbouring entry (E–NE) (**a**) and average distance between each accessions and its nearest entry in the core (A–NE) (**b**) against the size of core collection for bean data set. The bean data set was split into two halves with one half used to form collection and the other half used for evaluation of the core. Target (E–NE and A–NE) values are the maximum (E–ENE) or minimum (A–NE) possible values for each criterion for the half of the data used for evaluation (evaluation set), while actual (E–ENE and A–NE) values are obtained from a core collections that were using one half (training set) and evaluated using the quality evaluation half of the data (evaluation set)

It is clear from Fig. 9 that major differences may occur between the unknown value we intend to optimise (*Target*) and the actual value obtained when the core is formed using training set and evaluated using another set of data (*Actual*). Although the core collections obtained by optimising both E–NE and A–NE performed better than random sampling in capturing unknown diversity, the differences are quite small (5–15 % for E–NE and 1–5 % for A–NE). A similar result was also observed with the coconut data (see Electronic Supplementary Material: Appendix 2). Ronfort et al. (2006) found very little gain in the total number of alleles captured using the H and M strategy (Schoen and Brown, 1995) over random sampling when evaluation was done using a different set of data. The H strategy seeks to maximise the total number of alleles in the core collection by sampling accessions from groups in proportion to their within-group genetic diversity. On the other hand, the M strategy examines all possible core collections and singles out those that maximise the number of observed alleles at the marker loci. Their (Ronfort et al. 2006) major explanation was that the set of inbred lines used in the study had no redundancy, leaving little room for optimisation to improve the results over and above random sampling. The relatively small gain in our case is probably due to limited size (number of markers) and questionable quality of the data. For a data set with limited structure, we expect little gain by minimising A–NE compared to random sampling and this could explain the small difference observed in the common bean data, i.e. splitting the common bean data into two sets weakened the group structure of the data and thus resulting in very little gain.

For both crops the correlation between distance matrices formed by the two halves (core and evaluation) of the data was not very high, i.e. bean (0.79) and coconut (0.63). It is therefore not very surprising that when core collections are created using one half of the data set they perform rather poorly when evaluated with the other half of the data set.

## Conclusions and recommendations

A critical examination of the different methods for the evaluating the quality of core collections used in the literature shows that the choices of criteria for evaluating core collections are sometimes made arbitrarily, resulting in false conclusions regarding the quality of core collections and the methods to select them. The criterion of choice for evaluating the quality of core collections should be determined by the objectives or type of the core collection. If the core collection is made to represent the accessions in the collection (CC–I), the evaluation criterion should reflect that, and a criterion such as the A–NE we proposed in this paper should be used. If the core is to represent the range of genotypes and/or phenotypes in the collection (CC–X), a criterion such as the E–NE should be used. In addition, we stress that whenever possible or appropriate, the evaluation of core collections should be based on data that have not been used for the selection of the accessions for the core collection. When the core collection is intended for a specific user, the quality will have to be determined in terms of fitness-for-use such as the ease with which certain groups of material can be used or the likelihood of finding traits of interest.

In summary, we introduced two genetic distance-based criteria (A–NE and E–NE) for evaluating the quality of core collections. We strongly recommend distance-based criteria mainly for two reasons: (a) they combine information from all traits simultaneously, instead of using one trait at a time as most of the evaluation criteria used in literature do; (b) they are intuitive, easy to interpret and relate to the concept of representation of genetic diversity. These two newly proposed distance-based criteria are suitable for evaluating the two important types of core collections (CC–I and CC–X). These evaluation criteria can also be used as optimisation criteria when creating the core collections.

## Electronic supplementary material

Below is the link to the electronic supplementary material.

Supplementary material 1 (DOC 44 kb)
