# Supplementary material for: Quality of core collections for effective utilisation of genetic resources review, discussion and interpretation
Source: Theor Appl Genet. 2012 Sep 15;126(2):289–305. doi: 10.1007/s00122-012-1971-y (PMC3555244; doi:10.1007/s00122-012-1971-y)
Supplement: Supplementary file 1 — Supplementary material 1 (DOC 44 kb) [file 122_2012_1971_MOESM1_ESM.doc]

**Appendix 1: Description of Kullback-Liebler distance**

In probability theory and information theory, the Kullback–Leibler distance (KL) is a non-symmetric measure of the difference between two probability distributions *P* and *Q* (Kullback and Leiber, 1951). For two probability distributions P and Q, KL distance is defined as

$$K(P,Q)=E_{p}\left[\log\left(\frac{Q}{P}\right)\right]$$

where $E_{p}\left[.\right]$ indicates the expectation value with respect to the probability distribution *P*(the expectation is evaluated with respect to distribution *P*).

Typically *P* represents the "true" distribution of data, observations, or a precise calculated theoretical distribution. The measure *Q* typically represents a theory, model, description, or approximation of *P*. KL is always non negative and is zero only if the two distributions are identical. In core collection application, the distribution of a particular trait in the whole collection represents true distribution (P) which is approximated by the distribution of the trait in the core collection. KL distance would therefore be a suitable criterion for evaluating core collections selected for representing the distribution of the traits in the whole collection.

For normally distributed variables, KL distance can be calculated for univariate as well as multivariate data. For two multivariate normal densities KL is an explicit function of only their covariance (correlation) matrices ($\Sigma_{1}$ and$\Sigma_{2}$) and the only necessary condition is that the two covariance matrices be positive definite (Tumminello et al 2007, Chen et al, 2008). Given two probability density functions $P\left(\Sigma_{1},X\right)$ and $P\left(\Sigma_{2},X\right)$ KL is defined as

$K\left(P\left(\Sigma_{1},X\right),P\left(\Sigma_{2},X\right)\right)=\frac{1}{2}\left[\log\left(\frac{\left|\Sigma_{2}\right|}{\left|\Sigma_{1}\right|}\right)+tr\left(\Sigma_{2}^{-1}\Sigma_{1}\right)-n\right]$,

where n is the dimension of the space spanned by the X variable and $\left|\Sigma\right|$ indicates the determinant of $\Sigma$.

There are several other distance based criteria that can be used to compare the two distribution (example: Kolmogorov-Sminov test; Anderson-Darling distance)

Appendix 2

**Fig 10**: Plot of average distance between an entry and the nearest neighbouring entry (E-NE) (A) and average distance between each accessions and its nearest entry in the core (A-NE) (B) against the size of core collection for bean data set. The coconut data set was split into two halves with one half used to form collection and the other half used for evaluation of the core. Target (E-NE and A-NE) values are the maximum (E-ENE) or minimum (A-NE) possible values for each criterion for the half of the data used for evaluation (*evaluation set*), while actual (E-ENE and A-NE) values are obtained from a core collections that were using one half (training set) and evaluated using the quality evaluation half of the data (evaluation set).
